# Paeoniflorin Ameliorates Metabolic Dysfunction-Associated Steatotic Liver Disease by SYK/SH3BP2 Signaling Pathway

**DOI:** 10.34133/research.1100

**Published:** 2026-02-02

**Authors:** Yuqing Liu, Jie Tao, Dongyu Tan, Feifan Zheng, Zhuoxuan Su, Jianfeng Yuan, Chunmei Zhu, Zhensen Zheng, Xiuteng Zhou, Duosheng Luo

**Affiliations:** ^1^ Guangdong Metabolic Diseases Research Center of Integrated Chinese and Western Medicine, Key Laboratory of Glucolipid Metabolic Disorder, Ministry of Education of China, Institute of Chinese Medicine, Guangdong Pharmaceutical University, Guangdong TCM Key Laboratory for Metabolic Diseases, Guangzhou 510006, China.; ^2^ State Key Laboratory for Quality Ensurance and Sustainable Use of Dao-di Herbs, National Resource Center for Chinese Materia Medica, China Academy of Chinese Medical Sciences, Beijing 100700, China.

## Abstract

Metabolic dysfunction-associated steatotic liver disease (MASLD) has emerged as the leading cause of chronic liver disease globally and constitutes an independent risk factor for cardiovascular disease and mortality. Paeoniflorin (PF), the primary active compound derived from the traditional Chinese herb *Paeonia lactiflora* Pall., demonstrates multiple pharmacological activities. However, its anti-MASLD mechanisms remain incompletely elucidated. This study revealed that PF markedly ameliorates MASLD pathology by reducing hepatic lipid accumulation, inflammation, and fibrosis; ameliorating insulin resistance and liver function parameters; modulating key lipid metabolism genes (acetyl-coA carboxylase [ACC], sterol regulatory element-binding protein 1 [SREBP1], peroxisome proliferator-activated receptor gamma [PPAR-γ], fatty acid synthase [FASN], carnitine palmitoyltransferase 1 [CPT1], peroxisome proliferator-activated receptor alpha [PPAR-α], adipose triglyceride lipase [ATGL], and cluster of differentiation 36 [CD36]); decreasing pro-inflammatory factors (interleukin-1β [IL-1β], IL-6, tumor growth factor-α [TGF-α], and monocyte chemoattractant protein-1 [MCP-1]); and suppressing hepatic fibrosis markers (alpha-smooth muscle actin [α-SMA], tissue inhibitor of metalloproteinases-1 [TIMP1], collagen type I alpha 1 chain [COL1α1], fibronectin 1 [FN1], platelet-derived growth factor receptor beta [PDGFRβ], and plasminogen activator inhibitor-1 [PAI-1]). Through integrated transcriptomics and pharmacological overexpression approaches, we identified the SYK/SH3BP2 signaling pathway as the crucial mechanism driving MASLD pathogenesis. PF effectively attenuated hepatic metabolic dysregulation, inflammation, and fibrotic activation through inhibition of this pathway. Our work provided the first evidence establishing the SYK/SH3BP2 signaling axis as a pivotal pathway in MASLD progression, unveiling novel therapeutic targets while furnishing a mechanistic foundation for PF’s potential application in MASLD treatment.

## Introduction

Metabolic dysfunction-associated steatotic liver disease (MASLD) represents the most common chronic liver condition globally, affecting approximately 38% of adults and constituting a major contributor to liver-related morbidity and mortality [1,2]. The disease spans a clinicopathological spectrum from simple steatosis to metabolic dysfunction-associated steatohepatitis (MASH), underscoring the intricate interplay between hepatic lipid accumulation and systemic metabolic dysregulation. Notably, patients with progressive MASLD—particularly those with MASH and fibrosis—face a markedly elevated risk of developing extrahepatic complications, including type 2 diabetes, chronic kidney disease, and certain extrahepatic malignancies [3]. Therefore, elucidating the pathogenesis and identifying effective therapeutic strategies for MASLD are urgently needed.

Currently, the THR-β agonist Resmetirom is the only Food and Drug Administration-approved pharmacotherapy for MASH with moderate to advanced fibrosis (defined as stage F2 to F3 fibrosis), demonstrating efficacy in reducing hepatic fat, inflammation, and fibrosis with a favorable safety profile [4,5]. Nonetheless, drug development for MASLD remains challenging due to the disease’s multifactorial pathogenesis, variability in clinical trial outcomes, and stringent safety requirements. Consequently, the identification of effective and safe therapeutic agents for MASLD remains a substantial challenge.

**Fig. 1.**
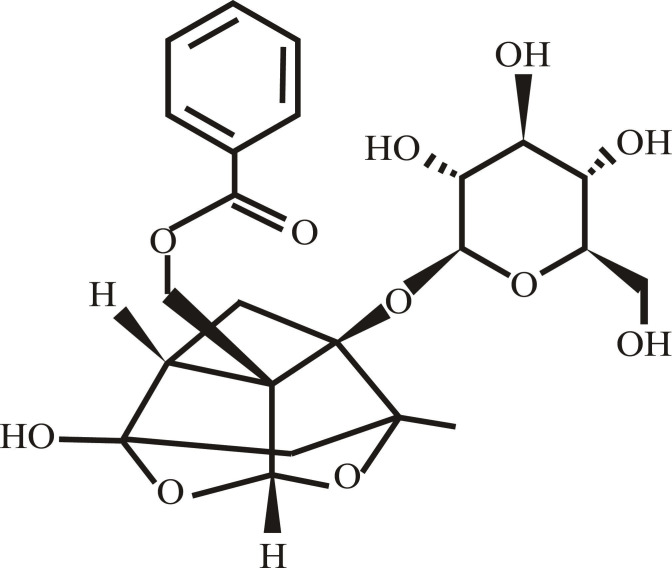
Chemical structure of PF.

Natural products derived from traditional Chinese medicine (TCM) represent a promising source of therapeutic candidates. For instance, herpetrione has been identified as a novel nuclear receptor ligand with potential anti-NASH activity [6]. Similarly, *Paeonia lactiflora* Pall., a key component in liver-protecting TCM formulas, has shown clinical benefits in alleviating MASLD symptoms, supporting its translational relevance. Its principal active constituent, paeoniflorin (PF) (Fig. 1), exhibits anti-inflammatory [7], antioxidant [8], anti-hyperlipidemic [9], and hepatoprotective [10] properties. Accumulating studies have reported multiple preliminary hepatoprotective effects of PF, such as improving IR in NAFLD animal models by promoting fatty acid oxidation [11], regulating lipid metabolism and inhibiting lipid ectopic deposition [12], and exerting anti-inflammatory effects in MASH rats [13]. However, the existing studies on PF in the treatment of MASLD mostly focus on individual pathological stages (such as steatosis or inflammation), and its mechanisms against the full MASLD spectrum from steatosis, hepatitis to fibrosis, especially the blocking effect of long-term administration on disease progression [14,15], remain insufficiently characterized, thereby limiting its therapeutic potential.

Therefore, to systematically evaluate PF’s capacity to intervene in the key pathological triad of MASLD—steatosis, inflammation, and fibrosis—and to address its stage-spanning therapeutic potential, we employed a high-fat, high-fructose-glucose diet (HFFD)-induced mouse model that closely recapitulates the metabolic and histological features of human MASLD. Through integrated phenotypic, histopathological, and transcriptomic analyses, this study aims to determine whether PF confers protection across the MASLD spectrum and to explore the molecular pathways underlying these effects.

## Results

### PF ameliorated dyslipidemia and liver injury in HFFD-induced MASLD mice

**Fig. 2.**
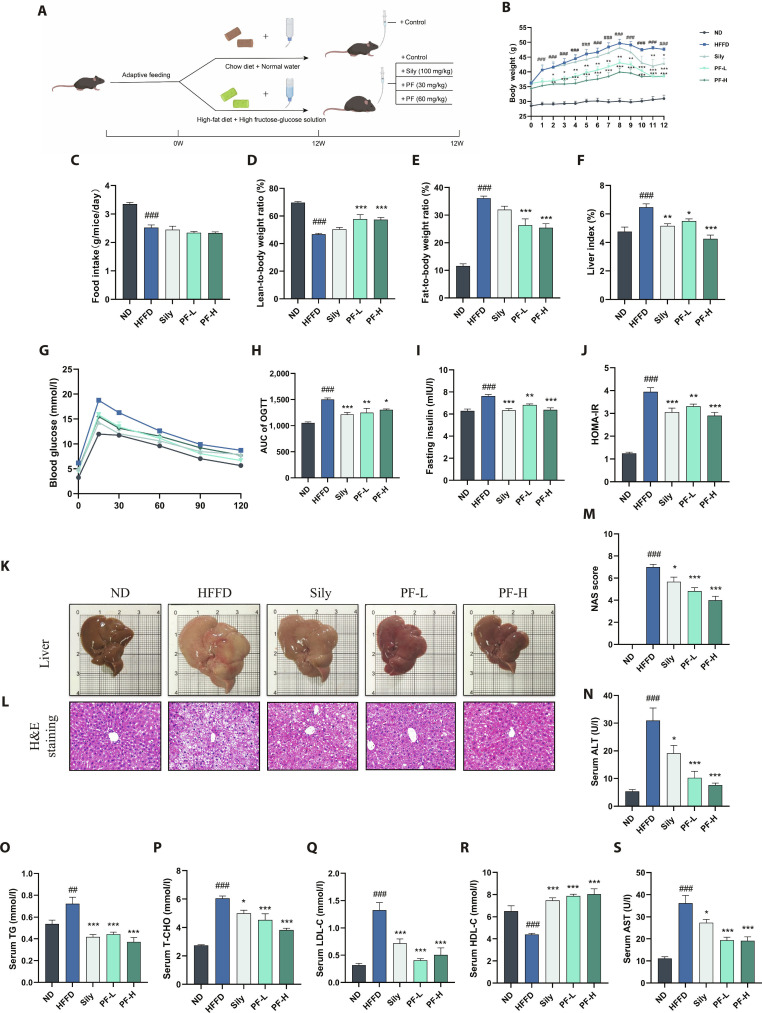
PF ameliorated dyslipidemia and liver injury in HFFD-induced MASLD mice. (A) Schematic representation. (B) Body weight. (C) Mouse food intake. (D and E) Mouse body composition analysis. (F) Mouse liver index. (G and H) OGTT. (I and J) Serum insulin content and insulin resistance index in mice. (K) Representative graphs of mouse liver gross morphology. (L and M) H&E staining results and NAS scores in mice (*n* = 3, scale bar = 50 μm). (N) Serum ALT levels. (O to R) Serum TG, TC, LDL-C, and HDL-C levels. (S) Serum AST levels. Data were presented as the mean ± SEM (*n* = 6). ^##^*P* < 0.01, ^###^*P* < 0.001 versus the ND group; *P < 0.05, **P < 0.01, ***P < 0.001 versus the HFFD group.

After 12 weeks of HFFD feeding and drug intervention (Fig. 2A), as shown in Fig. 2B to F, HFFD group mice had higher body weights, lower food intake, higher fat content, lower lean meat content, and higher liver index than ND group mice. PF intervention did not affect food intake but decreased fat content, increased lean meat content, and lowered liver index. Elevated lipids and aspartate aminotransferase (AST)/alanine aminotransferase (ALT) levels indicated metabolic imbalance and liver damage. To test PF’s protective effect on MASLD mice, blood lipids and liver injury markers were measured (Fig. 2N to S). Compared to ND group mice, HFFD group mice had higher total cholesterol (TC), triglycerides (TG), low-density lipoprotein cholesterol (LDL-C), AST, ALT, and lower high-density lipoprotein cholesterol (HDL-C). PF and Sily treatment improved these indices. Specifically, PF at different doses showed a dose-dependent effect on TG, TC, HDL-C, and ALT, but not on LDL-C and AST. Overall, PF effectively improved lipid profiles and liver function in MASLD mice.

Representative livers from each group are shown in Fig. 2K and L. ND group mice had reddish, small livers with smooth, dense surfaces and well-arranged hepatocyte–hepatic cords, no obvious vacuoles. HFFD group mice had larger, whitish livers with rough surfaces and yellow lipid droplet mottling. Their hepatocytes were disorganized, with severe lipid vacuoles, hepatocyte ballooning, lymphocyte infiltration, and nuclear consolidation. After Sily, PF-L, and PF-H intervention, liver size decreased, especially in PF-L and PF-H groups. Livers in these 2 groups had near-normal surface and color, with remarkably reduced vacuoles, balloon-like lesions, and inflammatory cell infiltration. NAFLD Activity Scores (NASs) (Fig. 2M) showed that Sily and PF effectively relieved liver pathology in HFFD-induced MASLD mice.

As shown in Fig. 2G and H, in the oral glucose tolerance test (OGTT) experiment, blood glucose values of mice in all groups peaked at 15 min. The HFFD group had higher blood glucose levels and reduced glucose tolerance than the other groups. The Sily, PF-L, and PF-H groups all substantially reduced the area under the curve (AUC) of the blood glucose curves. Figure 2I and J present the insulin resistance results, showing that the HFFD group had remarkably higher serum insulin levels and insulin resistance indices, which were substantially lowered in the Sily group and after different doses of PF intervention. In summary, both Sily and PF can remarkably enhance glucose tolerance, reduce serum insulin levels, and alleviate insulin resistance in MASLD mice.

### PF alleviated lipid metabolism disorders induced by HFFD in MASLD mice

**Fig. 3.**
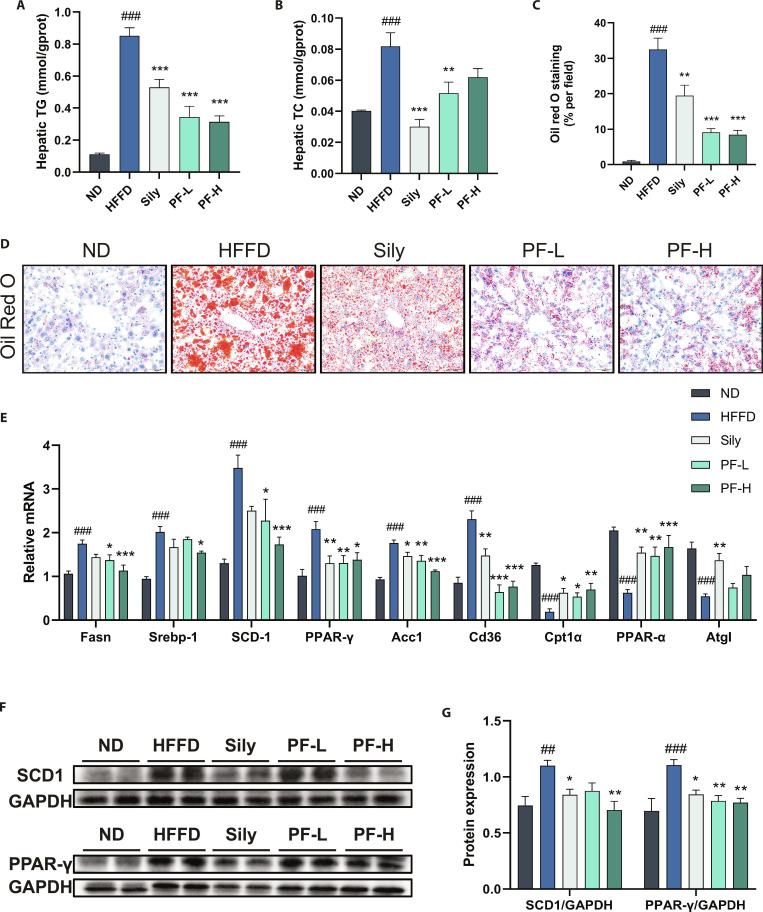
PF alleviated lipid metabolism disorders induced by HFFD in MASLD mice. (A and B) Hepatic TG and TC levels. (C and D) ORO staining and quantitative results (*n* = 3, scale bar = 50 μm). (E) mRNA levels in liver. (F and G) Western blot detection of hepatic SCD1 and PPAR-γ protein expression. Data were presented as the mean ± SEM (*n* = 6). ^##^*P* < 0.01, ^###^*P* < 0.001 versus the ND group; *P < 0.05, **P < 0.01, ***P < 0.001 versus the HFFD group.

Oil Red O (ORO) stain was used to explore PF’s impact on hepatic steatosis in MASLD mice (Fig. 3C and D). HFFD group mice showed numerous orange–red lipid droplets and severe hepatic lipid accumulation, with a significantly higher ORO-stained area than the ND group. After interventions with Sily and different PF doses, the stained area decreased substantially and liver TG and TC were notably reduced (Fig. 3A and B), indicating that both Sily and PF could markedly reduce lipid accumulation in MASLD mouse livers. As shown in Fig. 3E, the mRNA levels of genes related to hepatic lipid metabolism, such as fatty acid synthesis (Acc, Srebp-1c, Scd1, Ppar-γ, and Fasn) and fatty acid transport (Cd36), were remarkably up-regulated in the HFFD group, while fatty acid oxidation-related genes (Cpt1α, Ppar-α, Atgl) were substantially down-regulated. After the intervention of Sily and PF, the mRNA expression of the above-mentioned were reversed. Additionally, the expression levels of lipid synthesis-related proteins SCD1 and PPAR-γ in the liver tissues of HFFD-induced MASLD mice were remarkably higher than those in the ND group, but the intervention of Sily and PF reversed this up-regulation. These results indicated that PF can help maintain hepatic lipid metabolism homeostasis by inhibiting lipid synthesis and regulating fatty acid transport and oxidation.

### PF inhibited HFFD-induced inflammation and oxidative stress in MASLD mice

**Fig. 4.**
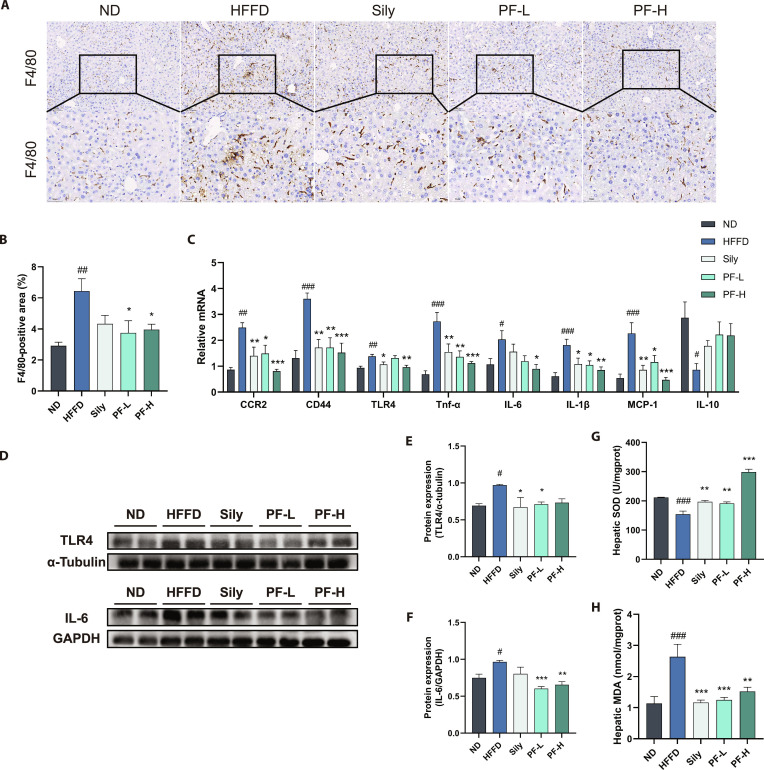
PF inhibited HFFD-induced inflammation and oxidative stress in MASLD mice. (A and B) Representative images and quantification of F4/80 immunohistochemical staining (*n* = 3). (C) Inflammation-related genes Ccr2, Cd44, Tlr4, Il-6, Il-1β, Tnf-α, Mcp-1, and Il-10 mRNA expression levels. (D to F) Western blot of TLR4, Il-6 protein expression, and its quantification. (G) Liver SOD activity. (H) Liver MDA content. Data were presented as the mean ± SEM (*n* = 6). ^#^*P* < 0.05, ^##^*P* < 0.01, ^###^*P* < 0.001 versus the ND group; *P < 0.05, **P < 0.01, ***P < 0.001 versus the HFFD group.

Inflammatory response is a notable pathological feature in MASLD progression, driving dysregulation of hepatic immune homeostasis and exacerbating hepatocyte injury through sustained inflammatory cytokine production. F4/80, a highly glycosylated cell-surface glycoprotein, serves as a macrophage surface marker in mature mouse macrophages. Immunohistochemical staining for F4/80 (see Fig. 4A and B) showed a significant increase in F4/80-positive cells in the liver tissues of the HFFD group. This increase was substantially reduced following the intervention with Sily and PF, indicating that both agents inhibited inflammatory infiltration in MASLD mice. As shown in Fig. 4C, mRNA levels of pro-inflammatory cytokines (Ccr2, Cd44, Tlr4, Il-6, Il-1β, Tnf-α, and Mcp-1) were significantly elevated while anti-inflammatory Il-10 was reduced in HFFD group livers versus the ND group. After intervention with Sily and different doses of PF, expression levels of Ccr2, Cd44, Tlr4, Il-6, Tnf-α, Il-1β, and Mcp-1 mRNA were substantially decreased, with Il-10 expression increased. Analysis of inflammation-related proteins (Fig. 4D to F) demonstrated that Sily and PF doses reversed elevated TLR4 and interleukin-6 (IL-6) expression, suggesting alleviation of hepatic inflammation in MASLD mice. Additionally, hepatic superoxide dismutase (SOD) activity was remarkably lower and malondialdehyde (MDA) levels were substantially higher in the HFFD group versus ND controls; conversely, the Sily, PF-L, and PF-H groups exhibited significantly higher SOD activity and lower MDA levels than the HFFD group (Fig. 4G and H), indicating that both Sily and PF enhance SOD activity, protect against oxidative damage, inhibit lipid peroxidation, and mitigate HFFD-induced oxidative stress.

### PF ameliorated HFFD-induced hepatic fibrosis progression in MASLD mice

**Fig. 5.**
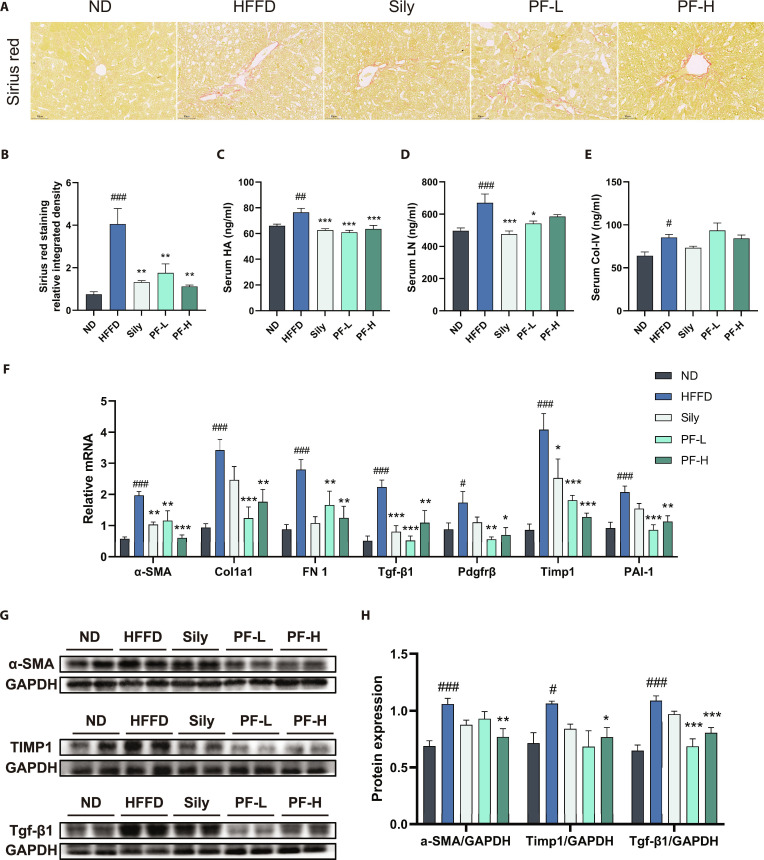
PF ameliorated HFFD-induced hepatic fibrosis progression in MASLD mice. (A and B) Representative images and quantification of hepatic Sirius scarlet staining. (*n* = 3, scale bar = 50 μm). (C to E) Serum fibrosis factor HA, LN, and Col-IV levels. (F) Liver mRNA levels of *α-SMA, Timp1, Tgf-β1, Col1a1, Fn1*, *Pdgfrβ*, and *PAI-1*. (G and H) Western blot of α-SMA, TIMP1, and TGF-β1 protein expression and their quantification. Data were presented as the mean ± SEM (*n* = 6). ^#^*P* < 0.05, ^##^*P* < 0.01, ^###^*P* < 0.001 versus the ND group; *P < 0.05, **P < 0.01, ***P < 0.001 versus the HFFD group.

Fibrosis represents a major driver of cardiovascular complications, malignancy, and mortality, constituting a prevalent pathological process in MASLD. Sirius red staining analysis of liver tissues across all mouse groups revealed that HFFD group mice exhibited significant fibrotic foci with substantially increased red collagen fibers compared to the ND group. Interventions with Sily and varying PF doses substantially reduced red collagen fiber deposition area (Fig. 5A and B). Serum fibrosis markers HA, LN, and Col-IV—commonly used to evaluate liver fibrosis progression—were remarkably elevated in the HFFD group versus ND controls (Fig. 5C to E). Following Sily and PF interventions, serum HA and LN levels were substantially reduced, with Col-IV also demonstrating a decreasing trend. Furthermore, mRNA levels and protein expression of fibrosis-related genes are shown in Fig. 5F to H: compared to ND, HFFD group mice exhibited significant up-regulation in mRNA/protein levels of α-SMA, Timp1, and Tgf-β1, along with increased mRNA expression of Col1a1, Fn1, Pdgfrβ, and PAI-1. Sily and PF interventions significantly reversed the up-regulation of α-SMA, Timp1, and Tgf-β1 mRNA/protein expression, and down-regulated mRNA levels of Col1a1, Fn1, Pdgfrβ, and PAI-1. These results demonstrate that PF inhibits fibrosis development in HFFD-induced MASLD mice.

### SYK/SH3BP2 pathway mediated PF’s therapeutic effects on MASLD: Evidence from RNA sequencing

**Fig. 6.**
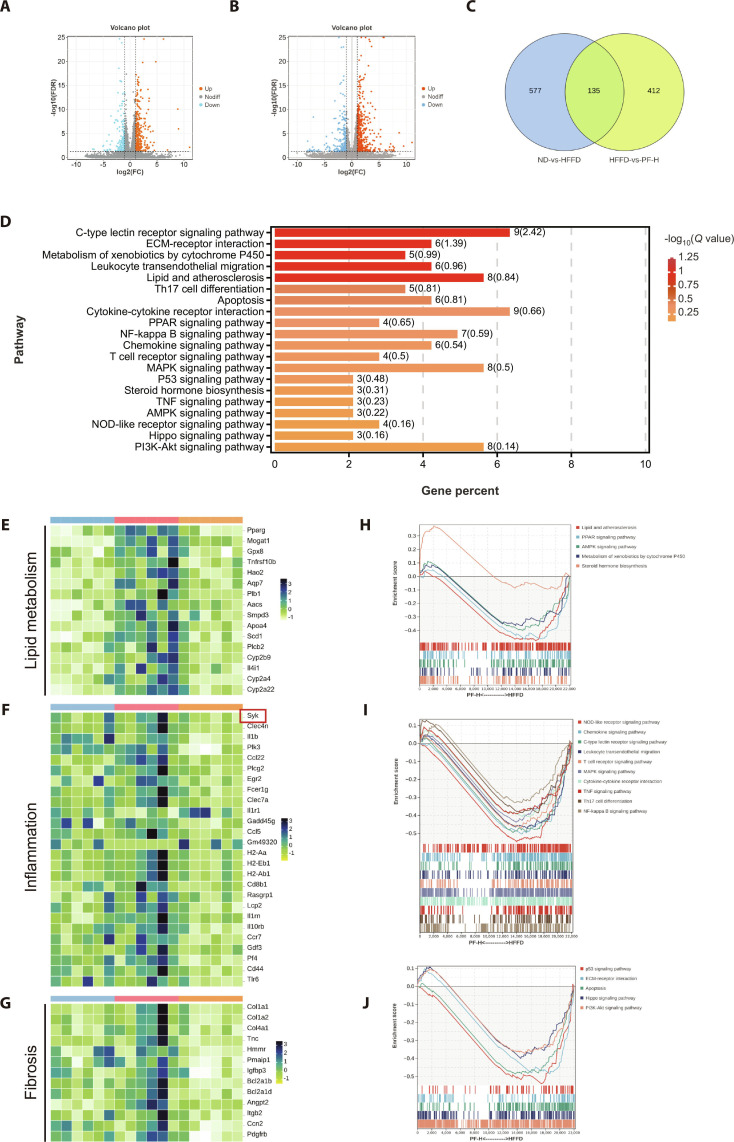
SYK/SH3BP2 pathway mediated PF’s therapeutic effects on MASLD: evidence from RNA sequencing. (A) Volcano plot of genetic statistics of liver samples from ND and HFFD groups. (B) Volcano plot of gene statistics of liver samples from HFFD and PF-H groups. (C) Differential gene Wayne plots of liver samples from ND, HFFD, and PF-H groups. (D) KEGG pathway enrichment analysis (top 20 enriched pathways). (E to G) Heatmap of gene expression. (H to J) GSEA pathway enrichment analysis. Data were presented as the mean ± SEM (*n* = 6).

Based on the above findings demonstrating the protective effects of PF against MASLD, transcriptomic sequencing was further employed to explore the underlying molecular mechanisms. Differentially expressed genes (DEGs) across sample groups are presented in Fig. 6A to C. Volcano plots illustrate the global distribution of DEGs between the ND and HFFD groups, and between HFFD and PF-H groups, with blue indicating substantially down-regulated genes and orange–red denoting remarkably up-regulated genes. Venn analysis identified 135 intersecting DEGs from ND-vs.-HFFD and HFFD-vs.-PF-H comparisons. Kyoto Encyclopedia of Genes and Genomes (KEGG) enrichment analysis (Fig. 6D) revealed that PF intervention primarily enriched pathways related to C-type lectin signaling and ECM remodeling. Conversely, inflammation-related pathways (C-type lectin, Toll-like receptors, and NF-κB) and metabolism-related pathways (PPAR and AMPK) showed significant down-regulation. DEGs were thus categorized into 3 domains: lipid metabolism (Fig. 6E), inflammation (Fig. 6F), and fibrosis (Fig. 6G). PF reduced expression of relevant genes in MASLD mouse livers and markedly suppressed SYK—a key inflammation regulator. Gene Set Enrichment Analysis (GSEA) of HFFD-vs.-PF-H DEGs demonstrated significant down-regulation in lipid metabolism (Fig. 6H), inflammatory response (Fig. 6I), and fibrosis-related pathways (Fig. 6J) following PF intervention, indicating comprehensive suppression of these pathogenic signaling axes in MASLD livers.

Given that the primary pharmacological action of PF is anti-inflammatory, and that mechanistic studies in this area are more abundant than those on lipid metabolism or fibrosis, we focused on inflammation-related gene sets (Fig. 6F). After excluding mouse-specific genes and those with modest differential expression in MASLD, 6 candidate human genes (CD44, SYK, FCER1G, PLCG2, CCL5, and IL1RN) were identified. Molecular docking showed that although CD44 had the highest binding affinity with PF, it was excluded due to extensive prior study. Given that the binding affinity of SYK to PF is second only to that of CD44 (Fig. S1 and Table S1), studies on its regulatory role in the field of metabolic steatohepatitis have been reported in recent years, SYK was selected as the research target.

### PF inhibited SYK/SH3BP2 signaling pathway in HFFD-induced MASLD

**Fig. 7.**
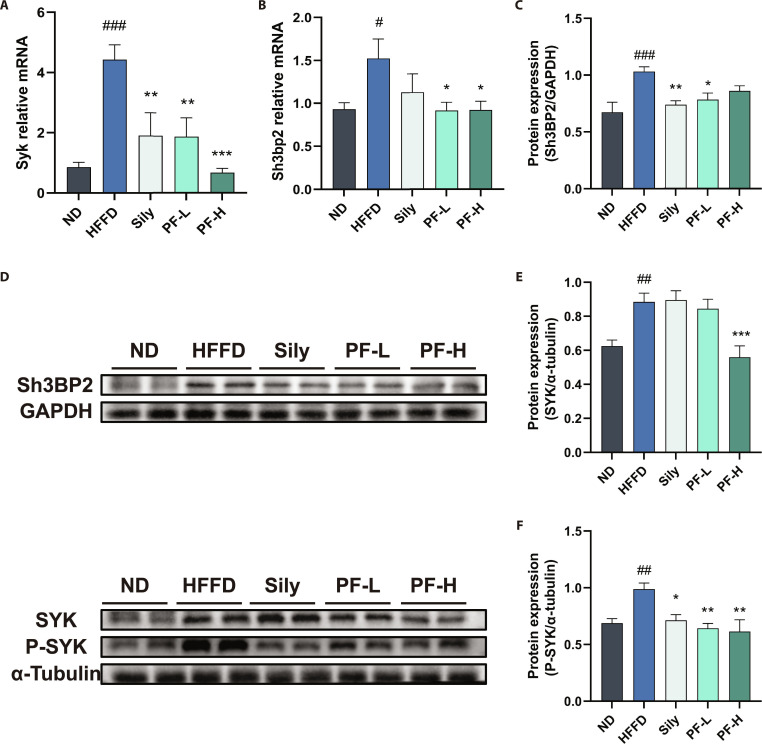
PF inhibited SYK/SH3BP2 signaling pathway in HFFD-induced MASLD. (A and B) Liver mRNA levels of *Syk* and *SH3BP2.* (C to F) Representative plots of the protein expression of liver Syk, P-Syk, and SH3BP2 with quantification. Data were presented as the mean ± SEM (*n* = 6). ^#^*P* < 0.05, ^##^*P* < 0.01, ^###^*P* < 0.001 versus the ND group; *P < 0.05, **P < 0.01, ***P < 0.001 versus the HFFD group.

Figure 7A and B show that hepatic mRNA levels of Syk and Sh3bp2 in HFFD group mice were higher than ND controls. PF intervention markedly down-regulated these expression levels. Previous studies indicate that phosphorylated SYK (p-SYK) exacerbates hepatic tissue injury and inflammatory responses and promotes steatosis progression [16]. Western blot analysis (Fig. 7C to F) revealed substantially elevated protein expression of P-SYK, SYK, and SH3BP2 in HFFD-group livers versus ND controls. PF intervention markedly down-regulated these protein levels, suggesting that PF attenuates hepatic inflammation by inhibiting SYK/SH3BP2 axis—thereby reducing inflammatory mediator release and suppressing immune cell activation/infiltration. Concurrently, this pathway inhibition may ameliorate lipid metabolism dysregulation and hepatic fibrosis, ultimately eliminating MASLD pathology.

### PF ameliorates dysregulated hepatic lipid metabolism and inflammation in MASLD via SYK/SH3BP2 pathway inhibition in vitro

**Fig. 8.**
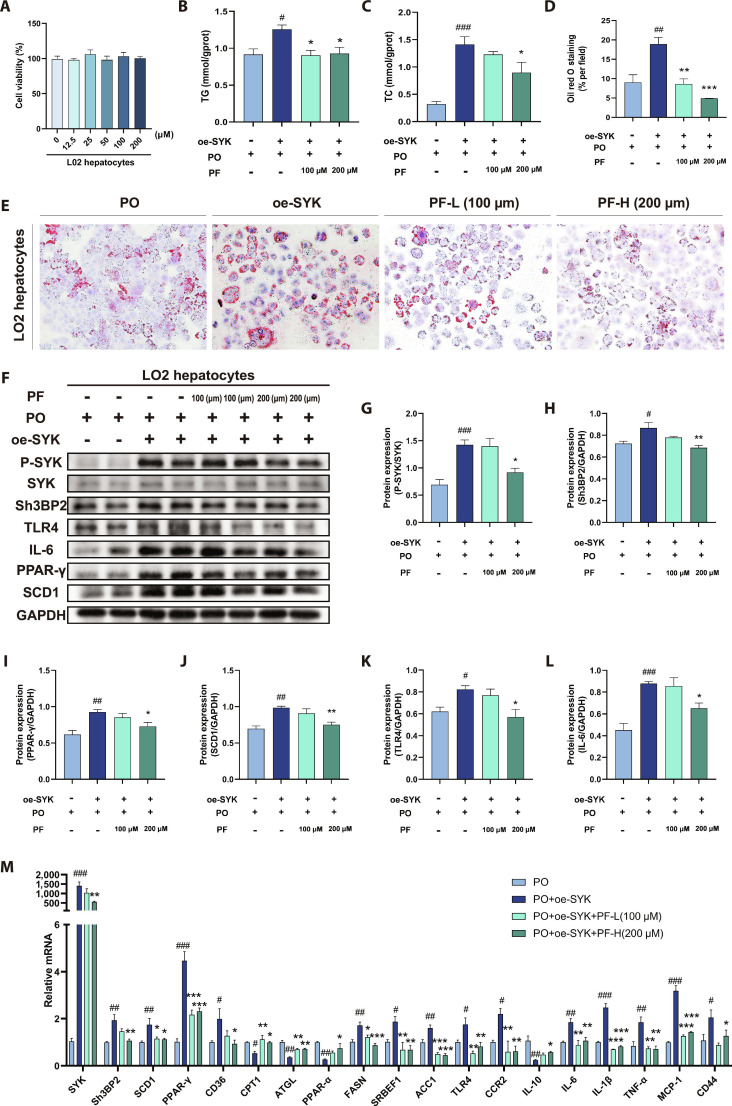
PF ameliorated dysregulated hepatic lipid metabolism and inflammation in MASLD via SYK/SH3BP2 pathway inhibition in vitro. (A) LO2 cell viability (*n* = 3). (B and C) LO2 cell TG and TC content (*n* = 3). (D and E) Representative graphs and quantification of LO2 cell ORO staining (*n* = 3, scale bar = 200 μm). (F to L) Western blot of P-SYK, SYK, SH3BP2, TLR4, IL-6, PPAR-γ, and SCD1 protein expression and their quantification. (M) *SYK, SH3BP2, ACC, SREBP-1C, SCD1, PPAR-γ, CD36, CPT1α, PPAR-α, ATGL, FASN, CCR2, CD44, TLR4, IL-6, IL-1β, TNF-α, MCP-1*, and *IL-6* mRNA levels. Data were presented as the mean ± SEM (*n* = 4). ^#^*P* < 0.05, ^##^*P* < 0.01, ^###^*P* < 0.001 versus the PO group; *P < 0.05, **P < 0.01, ***P < 0.001 versus the PO+oe-SYK group.

Cell counting kit-8 assay results (Fig. 8A) demonstrated no significant cytotoxicity of PF at different concentrations in LO2 cells. Interventions were subsequently performed at 100 and 200 μM PF [17]. Under SYK overexpression conditions, PO-induced LO2 cells exhibited significantly increased lipid deposition versus controls, evidenced by ORO staining showing increased lipid droplet number/size and elevated TC/TG content (Fig. 8B to E). However, 24-h PF treatment effectively reverses SYK-overexpression-induced lipid accumulation.

As shown in Fig. 8F to M, SYK overexpression substantially enhanced P-SYK and SH3BP2 protein expression while up-regulating SYK and SH3BP2 mRNA levels in PO-induced LO2 cells versus controls. Concomitantly, mRNA levels of lipid metabolism genes (ACC, SREBF1, SCD1, PPAR-γ, CD36, and FASN) were remarkably elevated, while CPT1, PPAR-α, and ATGL mRNA levels were reduced. Protein expression of SCD1 and PPAR-γ was substantially up-regulated. PF intervention effectively reversed these molecular alterations and restored lipid metabolic homeostasis.

Hepatic inflammatory response is a key driver of MASLD progression. SYK overexpression increased mRNA levels of pro-inflammatory genes (CCR2, CD44, TLR4, IL-1β, IL-6, tumor growth factor-α [TGF-α], and monocyte chemoattractant protein-1 [MCP-1]) while reducing anti-inflammatory IL-10 expression, disrupting inflammatory homeostasis (Fig. 8M). Correspondingly, TLR4 and IL-6 protein expression was markedly up-regulated (Fig. 8K and L). However, PF treatment reversed these molecular alterations, suppressing SYK/SH3BP2 pathway overactivation. This inhibition down-regulated pro-inflammatory gene/protein expression while promoting anti-inflammatory IL-10 production.

### PF alleviated MASLD fibrosis progression in vitro by inhibiting the SYK/SH3BP2 pathway

**Fig. 9.**
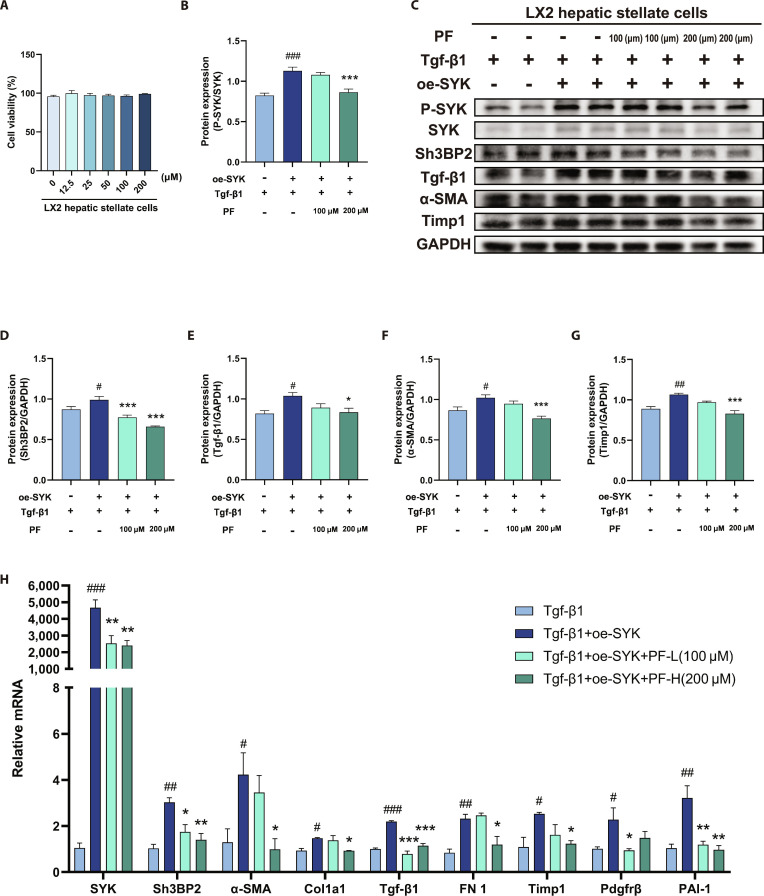
PF alleviated MASLD fibrosis progression in vitro by inhibiting the SYK/SH3BP2 pathway. (A) LX2 cell survival (*n* = 3). (B to G) Western blot of P-SYK, SYK, SH3BP2, α-SMA, TIMP1, and Tgf-β1 protein expression and their quantification. (H) mRNA levels of *SYK, SH3BP2, α-SMA, TIMP1, Tgf-β1, Col1a1, FN1, Pdgfrβ*, and *PAI-1*. Data were presented as the mean ± SEM (*n* = 4). ^#^*P* < 0.05, ^##^*P* < 0.01, ^###^*P* < 0.001 versus the Tgf-β1 group; *P < 0.05, **P < 0.01, ***P < 0.001 versus the Tgf-β1+oe-SYK group.

As shown in Fig. 9A, PF exhibited insignificant cytotoxicity to LX2 cells. These cells were selected for intervention with 100 and 200 μM concentrations of PF [17]. Under conditions of SYK overexpression, the protein expression levels of P-SYK and SH3BP2 were up-regulated (Fig. 9C and D), and the mRNA levels of SYK and SH3BP2 were also remarkably elevated compared to the control group induced by Tgf-β1 alone (Fig. 9E). Concurrently, the mRNA levels and protein expression of α-SMA (a hallmark protein for the activation of HSC), TIMP1 (which inhibits extracellular matrix degradation), and Tgf-β1 (a key pro-fibrotic cytokine) were substantially enhanced. Additionally, the mRNA levels of COL1a1, FN1 (both closely related to extracellular matrix synthesis), PDGFRβ, and PAI-1 were significantly up-regulated (Fig. 9). Thus, the up-regulation of these markers highlights the SYK/SH3BP2 pathway as a central mediator of TGF-β1-induced HSC activation and fibrosis. However, PF intervention markedly reversed the up-regulation of mRNA levels and protein expression of α-SMA, TIMP1, and Tgf-β1, and down-regulated the mRNA levels of COL1a1, FN1, PDGFRβ, and PAI-1. These results show that PF can effectively inhibit the SYK/SH3BP2 signaling pathway and counteract the Tgf-β1-induced fibrosis-related indicators, thereby mitigating the progression of MASLD fibrosis.

## Discussion

**Fig. 10.**
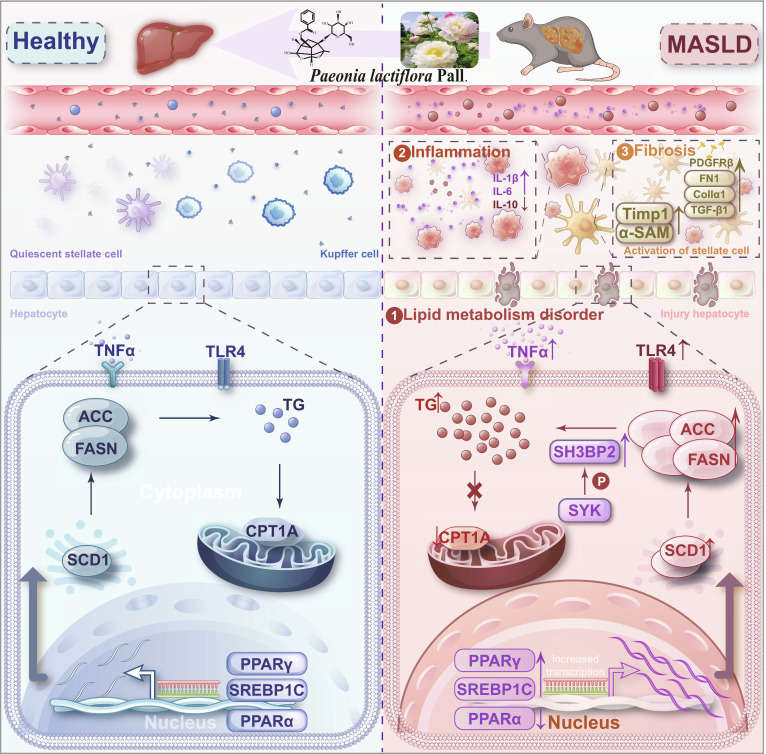
PF ameliorated MASLD via suppression of the SYK/SH3BP2 signaling pathway.

A major unresolved question in MASLD research is how metabolic dysregulation, inflammation, and fibrosis are mechanistically coordinated across disease stages rather than arising as isolated or sequential events [18]. The present study addresses this gap by identifying a shared immunometabolic signaling axis through which PF is associated with concurrent improvement of these 3 core pathological dimensions. Our findings support a model in which modulation of the SYK/SH3BP2 pathway is closely linked to multistage attenuation of MASLD pathology, thereby providing mechanistic insight beyond prior stage-restricted observations (Fig. 10).

By employing an HFFD mouse model [19,20], we were able to evaluate PF across the integrated pathological spectrum encompassing steatosis, immune activation, and fibrosis. Within this context, PF treatment was consistently associated with improved metabolic indices, reduced hepatic injury, and attenuation of fibrotic remodeling, supporting its potential as a multidimensional therapeutic agent (Fig. 2).

From a pharmacological and translational standpoint, currently, there is insufficient clinical research on PF and herbal medicines rich in PF, but TCM formulas that include *P. lactiflora* Pall. as a major component have been studied to some extent in the clinical treatment of MASLD (for example, Danggui Shaoyao San, Sini San, and Da Chai Hu Tang) [21]. In this study, PF was administered orally to reflect its established clinical use and physiological exposure. Although PF exhibits low oral bioavailability, largely due to limited intestinal permeability and efflux transport, this route preserves intestinal absorption and hepatic first-pass processing, which are integral to its in vivo disposition [22]. Previous studies indicate that PF undergoes intestinal and hepatic metabolism, potentially influencing its systemic exposure [18]. While specific PF-derived metabolites were not characterized here, their formation may contribute to overall pharmacological effects and warrants further investigation in future studies.

Thus, how does PF ameliorate the core pathological stages of MASLD? Recent advances in disease mechanisms highlight the importance of cell type-specific signaling pathways across MASLD progression [23]. Transcriptomic analysis of PF-treated MASLD models provided a directional clue for mechanistic investigation. Among the DEGs, we focused on spleen tyrosine kinase (SYK) based on its well-established involvement in liver diseases, the extensively documented anti-inflammatory properties of PF (with SYK being a key inflammation-associated kinase), and the favorable binding affinity observed between PF and SYK (Fig. S1 and Table S1). We therefore hypothesized that SYK represents a critical target through which PF exerts multistage protective effects in MASLD, and subsequently validated it (Figs. 6 and 7).

Specifically, SYK, a tyrosine kinase expressed in hepatocytes, Kupffer cells, and other hepatic cell types [24,25], is up-regulated in MASH patients and correlates with disease severity [26]. Its inhibition reduces neutrophil infiltration, immune cell activation, steatosis, and fibrosis [16,27,28]. SH3 structural domain binding protein 2 (SH3BP2) acts as an adaptor protein that, upon immunoreceptor engagement, is phosphorylated and recruits SYK to amplify downstream innate immune signaling [29,30]. Genetic ablation of either SYK or SH3BP2 in myeloid cells attenuates hepatic inflammation, underscoring their functional interdependence [31]. In HFFD-induced MASLD livers, we observed marked up-regulation of phosphorylated SYK, total SYK, and SH3BP2 at both transcriptional and protein levels, indicating sustained activation of the SYK/SH3BP2 signaling axis during disease progression. Consequently, the SYK/SH3BP2 axis operates across multiple hepatic cell types and disease stages to drive lipid accumulation, inflammation, and fibrogenesis, positioning it as a promising multifaceted target for MASLD therapy.

Consistent with prior studies showing that SYK inhibition ameliorates hepatic steatosis and restores lipid homeostasis [16,32]. SYK overexpression promoted hepatocellular lipid accumulation by up-regulating lipogenic genes (ACC, SREBF1, SCD1, PPAR-γ, FASN, and CD36) while suppressing fatty acid oxidation pathways (CPT1, PPAR-α, and ATGL). PF treatment countered these effects in SYK overexpression, consistent with its known suppression of de novo lipogenesis via SREBF1 inhibition [12,33]. Furthermore, PF down-regulated PPAR-γ expression, a key driver of lipid uptake and droplet formation, while restoring fatty acid oxidation [34]. Collectively, these effects reestablished intrahepatic lipid homeostasis (Figs. 3 and 8).

Beyond metabolism, SYK functions as a critical mediator of hepatic inflammation, particularly through macrophage recruitment and innate immune activation. SH3BP2 amplifies SYK-dependent inflammatory signaling, facilitating monocyte and neutrophil infiltration [35]. PF markedly suppressed SYK-driven pro-inflammatory mediators, including CCR2 (mediating inflammatory cell chemotaxis [36]), CD44 (enhancing leukocyte adhesion and macrophage polarization [37]), TLR4 (activating innate immunity [38]), NLRP3 inflammasome components (requiring SYK for IL-1β processing [39]), IL-6, TNF-α (amplifying inflammation [40,41]), and MCP-1 (recruiting monocytes [42]), while elevating anti-inflammatory IL-10 (Figs. 4 and 8).

A comprehensive single-cell atlas of mouse livers reveals the cellular activities and fates of several specific cell types (including hepatocytes, HSCs, endothelial cells, and Kupffer cells) in the liver. Ligand–receptor interactions between these cells promote the proliferation and activation of HSCs, leading to liver fibrosis [43]. Aberrant SYK signaling promotes HSC activation and extracellular matrix accumulation through TGF-β1-, PDGFRβ-, TIMP1-, FN1-, and PAI-1-dependent pathways [26,44–48] (Figs. 5 and 9). PF significantly attenuated these fibrogenic programs in both in vivo and in vitro models, mitigating TGF-β1-induced stellate cell activation [49]. Together, these findings position the SYK/SH3BP2 axis as an integrative immunometabolic node linking steatosis, inflammation, and fibrosis in MASLD (Figs. 2 to 9).

Conceptually, these findings extend the current understanding of MASLD pathogenesis. Rather than viewing steatosis, inflammation, and fibrosis as sequential events, our results support a network-based model in which SYK/SH3BP2 functions as an immunometabolic integrator operating across hepatic cell types and disease stages. This framework is consistent with emerging evidence that similar signaling hubs govern metabolic inflammation in disorders such as type 2 diabetes and atherosclerosis [50], suggesting that the relevance of SYK/SH3BP2 signaling may extend beyond MASLD.

This work presents several key innovations. First, we employed an HFFD to establish an MASLD mouse model that closely recapitulates human metabolic dysregulation (e.g., insulin resistance and hypertriglyceridemia) and the full histopathological spectrum of steatosis, inflammation, and fibrosis [19,51]. This model is more aligned with the 2020 international expert consensus definition of MASLD (which emphasizes the core role of metabolic dysfunction), providing more reliable evidence for the clinical translation of PF. Second, we first identified the SYK/SH3BP2 signaling axis as the core target through which PF exerts its anti-MASLD effects. RNA sequencing revealed PF-mediated down-regulation of SYK, and gain-of-function experiments in hepatocytes and HSCs confirmed that PF acts via inhibition of this pathway. This represents the first linkage between PF and the SYK/SH3BP2 axis, whose role in liver disease was recently established [31]. Third, we systematically demonstrated that PF concurrently ameliorates all 3 hallmark pathological processes in MASLD—lipid dysregulation, inflammation, and fibrosis—moving beyond prior studies focused on isolated stages [52].

Despite the promising findings, this study has several limitations. First, while the HFFD model recapitulates key metabolic and early fibrotic features of human MASLD, it does not fully replicate advanced fibrosis. Thus, the efficacy of PF in late-stage fibrotic MASLD remains to be evaluated. Second, although gain-of-function experiments in vitro support the role of the SYK/SH3BP2 axis, validation in cell-type-specific or inducible transgenic animal models would further strengthen the mechanistic evidence. Third, the precise molecular crosstalk between SYK and SH3BP2 in different hepatic cell types during MASLD progression warrants deeper investigation, including phosphoproteomic and spatial transcriptomic analyses. Finally, while PF shows favorable short-term tolerability, its long-term safety and pharmacokinetic profile require systematic assessment in chronic toxicity studies before clinical translation. These limitations highlight the need for further preclinical work to fully delineate the therapeutic potential and safety of PF in MASLD.

## Conclusion

In this study, we demonstrated the therapeutic effects of PF in MASLD mice and explored the underlying molecular mechanisms. PF markedly alleviated insulin resistance, liver function, and injury, through modulating the expression of related genes and blunting the pathways involved in lipid metabolism, inflammatory responses, and fibrogenesis, ultimately alleviating MASLD symptoms. Moreover, through integrated transcriptomic analysis and pharmacological overexpression experiments, we identified the SYK/SH3BP2 signaling axis as a critical driver of MASLD progression. Inhibition of this pathway by PF attenuated hepatic metabolic dysregulation, inflammatory activation, and fibrotic remodeling. Collectively, these findings provide the first evidence linking PF to the SYK/SH3BP2 axis in MASLD, highlight a previously underappreciated molecular pathway in disease pathogenesis, and offer a mechanistic basis for the potential development of PF as a therapeutic agent for MASLD.

## Materials and Methods

### Animal models establishment and drug administration

Male specific-pathogen-free C57BL/6 mice (6 weeks) were purchased from Zhuhai Baishitong Experimental Animal Technology Co., Ltd. (Animal License No. SYXK (Yue) 2022-0125). Experimental procedures were approved by the Guangdong Pharmaceutical University Laboratory Animal Ethics Committee (Approval No. GDPULAC2022247).

Mice were randomly divided into 5 groups: normal diet (ND), model (HFFD), low-dose PF (PF-L), high-dose PF (PF-H), and silymarin (Sily), with 6 mice per group. The ND group received normal diet (standard rodent maintenance feed provided by Guangdong Pharmaceutical University Laboratory Animal Center) (Table S3). HFFD, PF-L, PF-H, and Sily groups were fed a high-fat diet (Dietz Biotech, Wuxi) (Table S2). The ND group drank autoclaved water, while other groups received high fructose–glucose solution (23.1 g/L D-fructose + 18.9 g/L D-glucose) [19]. After 12 weeks of modeling, PF-L and PF-H groups received oral gavage of PF (30 and 60 mg/kg, dissolved in 0.5% CMC-Na at specified time points). The Sily group received silymarin (100 mg/kg silymarin). Both ND and HFFD groups were administered 0.5% CMC-Na. Gavage volume was 0.1 ml/10 g for 12 weeks. PF was purchased from Chengdu Manst Network Technology Co., Ltd.(China). Silymarin was obtained from MADAUS GmbH (Germany).

Overnight fasted mice were sacrificed after anesthesia with isoflurane. Blood samples were collected by retro-orbital bleeding and centrifuged at 3,500 rpm for 15 min at 4 °C to obtain serum. The samples were stored at −20 °C until assayed. Livers were obtained, frozen in liquid nitrogen, and stored at −80 °C for subsequent analysis.

### Oral glucose tolerance test

The OGT test was conducted in accordance with the protocol [53]. Following a 12-h fast (with water access), mice received 20% glucose solution. Blood glucose levels were measured using glucose test strips at 0, 15, 30, 60, 90, and 120 min post-administration. The AUC was calculated using GraphPad Prism 9.0.

### Assessment of insulin resistance

Serum fasting insulin (FINS) levels were detected by enzyme-linked immunosorbent assay (ELISA). Homeostasis model assessment - insulin resistance (HOMA-IR), an index of insulin resistance, was estimated by the formula: FINS × FBG/22.5. The FINS ELISA kit was supplied by Jiangsu Meimian (China).

### Biochemical analysis

Assay kits of TC (A111-2-1), TG (A110-1-1), LDL-C (A113-1-1), HDL-C (A112-2-1), ALT (C009-2-1), AST (C010-2-1), SOD (A001-3) activity, and MDA (A003-1-2) were purchased from Nanjing Jiancheng Bioengineering Institute. Serum hyaluronic acid (MM-0514M1), laminin (MM-016M1), and Col-IV (MM-45905M1) levels were measured with kits supplied by Jiangsu Meimian (China). All assays were performed according to manufacturers’ protocols.

### Histopathological analysis

The collected liver tissues were fixed in a 4% paraformaldehyde solution for 24 h. Hematoxylin–eosin (H&E) staining, ORO staining, and Sirius red staining were performed on 4-μm-thick fixed slices according to the protocol. Pathological images were captured with an Olympus microscope (BX53, Olympus, Japan), and the positive area was quantified with ImageJ software

### Liver histology assessment

Liver histology was evaluated using the NAS system. Histological parameters were assessed on H&E-stained liver sections from each group [54]. Individual scores were summed to generate the total NAS.

### Cell culture, transfection, and treatment

LO2 and LX2 cells were purchased from the Shanghai Cell Bank, Chinese Academy of Sciences and cultured in Dulbecco’s modified Eagle medium (C11995500BT, Gibco) supplemented with 1% antibiotics and 10% fetal bovine serum at 37 °C under a humidified atmosphere of 5% CO₂. A mixture of 1 mM oleic acid (O1008, Sigma) and 0.5 mM palmitic acid (2:1; P0500, Sigma) was used to treat cells for 12 h [55].

For SYK overexpression, LO2 or LX2 cells were transfected for 24 h with either SYK overexpression plasmid (K24L2148, Shanghai Genechem Co.,Ltd.) or empty vector using Lipofectamine 3000 (L3000015, Thermo Fisher Scientific).

LX2 cells or transfected LX2 cells were treated with 10 ng/ml TGF-β1 (HY-P7118, MCE) for 24 h [56]. PF-L and PF-H were established, with both treatment groups receiving corresponding concentrations of the drug for 24 h.

### ORO staining

Cells were stained with ORO following the manufacturer’s instruction (G1262, Solarbio). Firstly, the cells were seeded at a density of 5 × 10^5^ in 24-well plates. After cell attachment, transfections, modeling, and drug treatments, the cells were washed twice with PBS, and then fixed with ORO Fixative for 20 to 40 min. Then, samples were washed twice with PBS followed by staining with freshly prepared ORO dye for 30 min. Hematoxylin staining solution was applied to stain the cell nucleus. Mounted sections were imaged using an Olympus microscope and analyzed with ImageJ for lipid droplet area quantification.

### qRT-PCR

Trizol reagent (9108, Takara) was used to extract the total RNA from the liver tissues or cultured cells and extracted with chloroform, then precipitated with iso-propanol, washed with 75% ethanol, and dissolved in diethyl pyrocarbonate water. Total RNA was reverse transcribed to cDNA using a PrimeScript RT kit with gDNA Eraser (RR047A, Takara). A quantitative real-time polymerase chain reaction (qRT-PCR) was conducted by using a TB Green Premix Ex Taq II (RR820A) as directed by the manufacturer with the real-time PCR System (LightCycler480). Gene expression levels were calculated using the 2^−ΔΔCt^ method. Primer sequences were provided in the Supplementary Materials (Table S4).

### RNA sequencing analysis

Total liver RNA was extracted using TRIzol reagent (9108, Takara). RNA integrity and size distribution were assessed by agarose gel electrophoresis, with quality verification on an Agilent 2100 Bioanalyzer. Following PCR amplification, sequencing was performed on an Illumina HiSeq 2500 system (Gene Denovo Biotechnology Co.). DEGs were identified using a threshold of |fold change| ≥ 1.5 for all possible comparisons. Sequencing services were outsourced to Guangzhou GENE DENOVO Co., Ltd.

### Western blotting

Radioimmunoprecipitation assay lysis buffer was used to extracted total proteins. After quantifying protein concentration, samples were separated by sodium dodecyl sulfate–polyacrylamide gel electrophoresis and transferred to NC membranes (HATF00010, Merck Millipore, Germany). These membranes were blocked with the blocking solution at 37 °C for 1 h, then incubated with primary antibodies for 12 h at 4 °C. After TBST washes, membranes were incubated with secondary antibodies for 1 h at 37 °C. The antibodies used are as follows: SCD1 (2794T, CST, 1:1,000), PPAR-γ (66936-1-Ig, Proteintech, 1:2,000), IL-6 (12242T, CST, 1:1,000), TLR4 (66350-1-Ig, Proteintech, 1:3,000), TIMP1 (33502-1, SAB, 1:3,000), α-SMA (67735, Proteintech , 1:20,000), Tgf-β1 (26155, Proteintech, 1:1,000), α-tubulin (66031, Proteintech, 1:20,000), GAPDH (60004-1-IG, Proteintech, 1:6,000), Phospho-Syk (2710T, CST, 1:1,000), Syk (2712T, CST, 1:1,000), SH3BP2 (PA5-87739, Invitrogen, 1:1,000), Goat anti-Mouse IgG Secondary Antibody HRP (L3032, SAB, 1:5,000), and Goat anti-Rabbit IgG Secondary Antibody HRP (L3012, SAB, 1:5,000). Finally, imaging and analysis were performed using ECL Luminescence Reagent (MA0186, Meilunbio, China) with a chemiluminescence imaging system. Band intensities were quantified using ImageJ software.

#### Statistical analysis

Parametric data were analyzed using unpaired Student’s *t* tests for 2-group comparisons or one-way analysis of variance followed by Tukey’s post hoc test for multiple-group comparisons. Nonparametric data were analyzed using the Mann–Whitney *U* test. Data conforming to a normal distribution are presented as means ± standard errors of the mean (SEM) [57]. All statistical analyses were performed using GraphPad Prism 9.4.1. A *P* value < 0.05 was considered statistically significant.

## Data Availability

The data are freely available upon request.
