# Supplementary material for: Paeoniflorin Ameliorates Metabolic Dysfunction-Associated Steatotic Liver Disease by SYK/SH3BP2 Signaling Pathway
Source: Research (Wash D C). 2026 Feb 2;9:1100. doi: 10.34133/research.1100 (PMC12862135; doi:10.34133/research.1100)
Supplement: Supplementary 1 — Fig. S1 Tables S1 to S4 [file research.1100.f1.zip › SUPPLEMENTARY MATERIALS.docx]

SUPPLEMENTARY MATERIALS

Fig. S1. Molecular docking diagram of Paeoniflorin with six key targets#
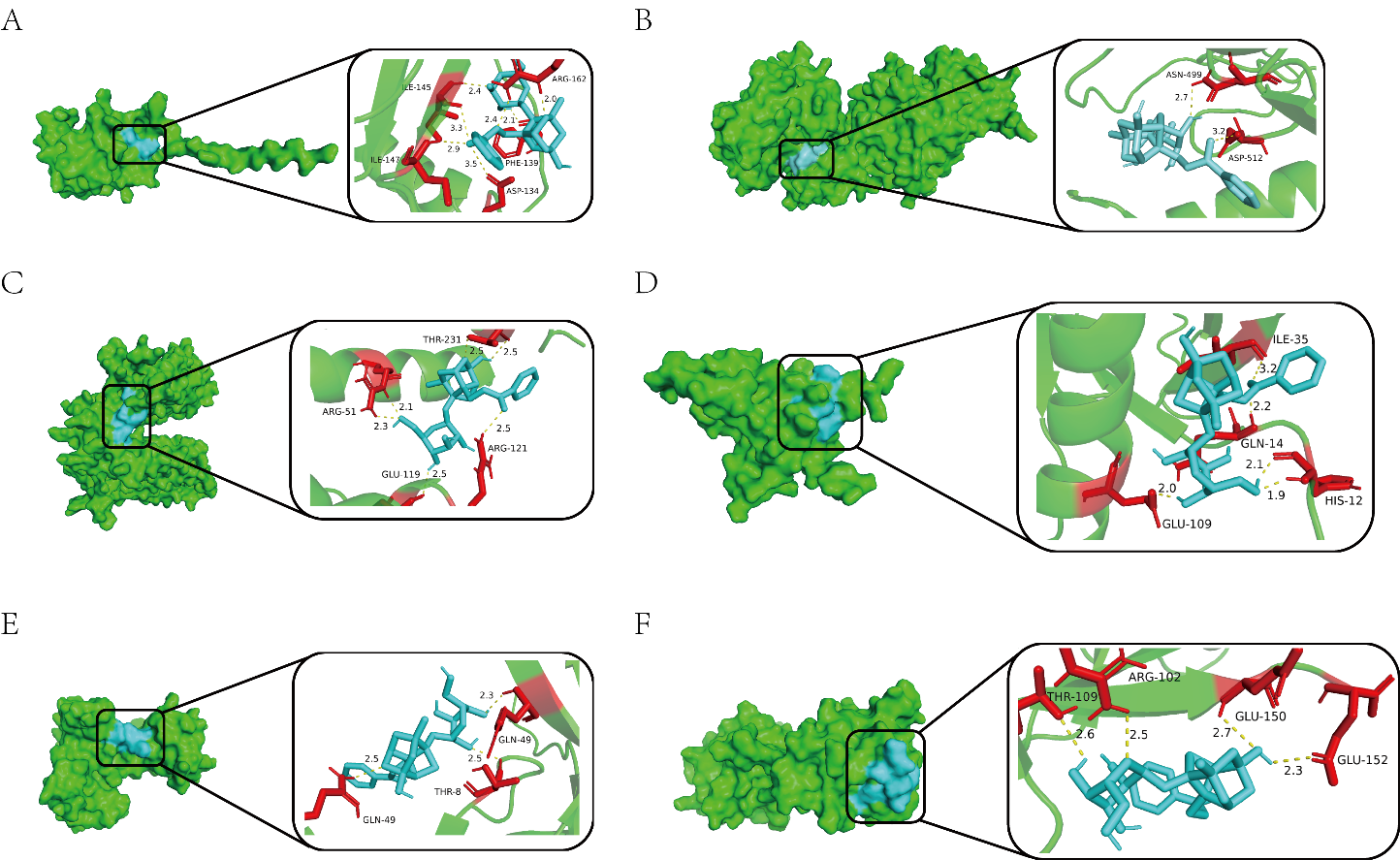


(A-F) Molecular docking diagram of CD44, SYK, FCER1G, PLCG2, CCL5, IL1RN with Paeoniflorin

Tables S1 to Molecular docking results of Paeoniflorin with six key targets#

| Ligand | Receptor | Affinity/kcal·mol^-1^ |
| --- | --- | --- |
| Paeoniflorin | CD44 | -7.3 |
|  | SYK | -7.1 |
|  | FCER1G | -6.9 |
|  | PLCG2 | -6.9 |
|  | CCL5 | -6.7 |
|  | IL1RN | -6.6 |

Tables S2 to High-fat diet (60 kcal% fat calories)#

| **Ingredient** | **Kcal/g** | **gram** | **kcal** |
| --- | --- | --- | --- |
| Casein | 4 | 200 | 800 |
| L-Cystine | 4 | 3 | 12 |
| Sucrose | 4 | 68.8 | 275 |
| Dyetrose | 4 | 125 | 500 |
| Lard | 9 | 245 | 2205 |
| Soybean oil | 9 | 25 | 225 |
| Cellulose | 0 | 50 | 0 |
| Mineral Mix #210088 | 1.6 | 10 | 16 |
| Calcium Carbonate | 0 | 5.5 | 0 |
| Dicalcium Phosphate | 0 | 13 | 0 |
| Potassium Citrate H2O | 0 | 16.5 | 0 |
| Vitamin Mix #300050 | 3.9 | 10 | 39 |
| Choline Bitartrate | 0 | 2 | 0 |
| Blue Dye | 0 | 0.05 | 0 |
| Total |  | 773.85 | 4072 |

| **Ingredient** | gram | kcal |
| --- | --- | --- |
| Protein | 26 | 20 |
| Carbohydrate | 26 | 20 |
| Fat | 35 | 60 |
| kcal/g | 5.26 | |

Tables S3 to Rodent maintenance feed#

| **Ingredient** | **gram** |
| --- | --- |
| Moisture | 76 |
| Protein | 215 |
| Fat | 42 |
| Fiber | 37 |
| Ash | 64 |
| Ca | 12.5 |
| P | 7.4 |
| **Total** | 453.9 |

Table S4. Primer sequences for qRT-PCR.

| **Primer** | **Sequence (5' to 3')** | **Origin** | **Length** |
| --- | --- | --- | --- |
| *Tnf-α*-F | CGC TCT TCT GTC TAC TGA ACT TCG G | Mouse | 25 |
| *Tnf-α*-R | GTG GTT TGT GAG TGT GAG GGT CTG | Mouse | 24 |
| *Il-6*-F | AGT TGC CTT CTT GGG ACT GA | Mouse | 20 |
| *Il-6*-R | TCC ACG ATT TCC CAG AGA AC | Mouse | 20 |
| *Il-1β*-F | CCG TGG ACC TTC CAG GAT GA | Mouse | 20 |
| *Il-1β*-R | GGG AAC GTC ACA CAC CAG CA | Mouse | 20 |
| *Mcp-1*-F | CTC TTC ACC TGC TCC ACT GC | Mouse | 20 |
| *Mcp-1*-R | TGG CTC AGC CAG ATG CAG T | Mouse | 19 |
| *ColⅠα1-F* | GCT CCT CTT AGG GGC CAC T | Mouse | 19 |
| *ColⅠα1-R* | CCA CGT CTC ACC ATT GGG G | Mouse | 19 |
| *Tgfβ1-F* | GTG GAA ATC AAC GGG ATC AG | Mouse | 20 |
| *Tgfβ1-R* | ACT TCC AAC CCA GGT CCT TC | Mouse | 20 |
| *α-SMA-F* | AGC CCA GCC TCT ACA TCT TT | Mouse | 20 |
| *α-SMA-R* | TCC TGG AAT GCT AAC AGG AG | Mouse | 20 |
| *FN-1-F* | GAC AGT TGG TCA CCC TGT TC | Mouse | 20 |
| *FN-1-R* | TGA CTT TCC TGC TCA AGG TC | Mouse | 20 |
| *Scd 1-F* | GCA AGC TCT ACA CCT GCC TCT T | Mouse | 22 |
| *Scd 1-R* | CGT GCC TTG TAA GTT CTG TGG C | Mouse | 22 |
| *Ppar-γ-F* | CCA TCG AGG ACA TCC AAG ACA ACC | Mouse | 24 |
| *Ppar-γ-R* | GGA GCA CCT TGG CGA ACA GC | Mouse | 20 |
| *Cd36-F* | TGA GAC TGG GAC CAT TGG TGA T | Mouse | 22 |
| *Cd36-R* | CCC AAG TAA GGC CAT CTC TAC CAT | Mouse | 24 |
| *Cpt1-F* | CTA CAT CAC CCC AAC CCA TAT T | Mouse | 22 |
| *Cpt1-R* | GAT CCC AGA AGA CGA ATA GGT T | Mouse | 22 |
| *Ppar-α-F* | TGC CTT CCC TGT GAA CTG AC | Mouse | 20 |
| *Ppar-α-R* | TGG GGA GAG AGG ACA GAT GG | Mouse | 20 |
| *Atgl-F* | GAG GAA TGG CCT ACT GAA CCA | Mouse | 21 |
| *Atgl-R* | GGC TGC AAT TGA TCC TCC TCT | Mouse | 21 |
| *FASN-F* | TTG ACG GCT CAC ACA CCT AC | Mouse | 20 |
| *FASN-R* | ACA GCC TGG GGT CAT CTT TG | Mouse | 20 |
| *ACC-F* | GTT CAG AGA GTT CAC CCA GCA | Mouse | 21 |
| *ACC-R* | AAC TAG GAA CGT AAG TCG CCG | Mouse | 21 |
| *Srebp-1C-F* | CCG AGA TGT GCG AAC TGG A | Mouse | 19 |
| *Srebp-1C-R* | ATA GGG GGC GTC AAA CAG G | Mouse | 19 |
| *TLR4-F* | ATG GCA TGG CTT ACA CCA CC | Mouse | 20 |
| *TLR4-R* | GAG GCC AAT TTT GTC TCC ACA | Mouse | 21 |
| *CD44-F* | CAC CTT GGC CAC CAC TCC TAA T | Mouse | 22 |
| *CD44-R* | TCA CAT GGG AGT CTT CAC TTG G | Mouse | 22 |
| **Primer** | **Sequence (5' to 3')** | **Origin** | **Length** |
| *CCR2-F* | GCC ATC ATA AAG GAG CCA TAC C | Mouse | 22 |
| *CCR2-R* | GTC TTT GCA GGC AGC TGA AC | Mouse | 20 |
| *Timp1-F* | ATC AGT GCC TGC AGC TTC TT | Mouse | 20 |
| *Timp1-R* | TCT GGT AGT CCT CAG AGC CC | Mouse | 20 |
| *Pdgfrb-F* | AGC CAG AAG TAG CGA GAA GC | Mouse | 20 |
| *Pdgfrb -R* | GGC AGT ATT CCG TGA TGA TG | Mouse | 20 |
| *PAI-1-F* | AAA TGG TGG CCC AAT AGC GA | Mouse | 20 |
| *PAI-1-R* | AAG CAA GCT GTG TCA AGG GA | Mouse | 20 |
| *Gapdh*-F | CAT CAC TGC CAC CCA GAA GAC TG | Mouse | 23 |
| *Gapdh*-R | ATG CCA GTG AGC TTC CCG TTC AG | Mouse | 23 |
| *SYK*-F | TTA GTG ATT CTG CCC GGC TC | Mouse | 20 |
| *SYK*-R | ACT TAG ACA GGA CAG CAC GC | Mouse | 20 |
| *SH3BP2*-F | GAA GCT TGG GAG CAA AGG CT | Mouse | 20 |
| *SH3BP2*-R | AAA TTC TGG GCA CCA ATG GC | Mouse | 20 |
| *Syk*-F | CGA GGG AAA GAA GTT CGA CAC G | Human | 22 |
| *Syk*-R | CCA GGC TTT GGG AAG GAG TAT G | Human | 22 |
| *Sh3bp2*-F | CTC CTA CCT GGA GCC TGA CT | Human | 20 |
| *Sh3bp2*-R | TGC ATC AGG GCA TCC TCA AG | Human | 20 |
| *TLR4*-F | CAC CCC GAT TCC ATT GCT TC | Human | 20 |
| *TLR4*-R | TTA GGA ACC ACC TCC GTG A | Human | 19 |
| *CCR2*-F | GGA GAA GTT CAG AAG CCT TTT TC | Human | 23 |
| *CCR2*-R | CCT TTT CCA CGA CCA TCG AG | Human | 20 |
| *CD44*-F | GAC AAC CAC AAG GAT GAC TGA | Human | 21 |
| *CD44*-R | GGA GTT GCC TGG ATT GTG CT | Human | 20 |
| *IL-10*-F | CGA GAT GCC TTC AGC AGA GT | Human | 20 |
| *IL-10*-R | GGC AAC CCA GGT AAC CCT TA | Human | 20 |
| *IL-6*-F | CCT TCT CCA CAA GCG CCT TC | Human | 20 |
| *IL-6*-R | AAG GCA GCA GGC AAC ACC A | Human | 19 |
| *IL-1β*-F | CCA AAC CTC TTC GAG GCA CA | Human | 20 |
| *IL-1β*-R | GGC TGC TTC AGA CAC TTG AG | Human | 20 |
| *TNF-α*-F | CTC GAA CCC CGA GTG ACA AG | Human | 20 |
| *TNF-α*-R | TCA GCT TGA GGG TTT GCT ACA | Human | 21 |
| *MCP-1*-F | TCT CGC CTC CAG CAT GAA AG | Human | 20 |
| *MCP-1*-R | GGC ATT GAT TGC ATC TGG CT | Human | 20 |
| *Scd 1*-F | TTC CCG ACG TGG CTT TTT CT | Human | 20 |
| *Scd 1*-R | AGC CAG GTT TGT AGT ACC TCC | Human | 21 |
| *Ppar-γ*-F | CCA GAA GCC TGC ATT TCT GC | Human | 20 |
| *Ppar-γ*-R | GTG TCA ACC ATG GTC ATT TCG TT | Human | 23 |
| *Cd36*-F | TTG GGA AAG TCA CTG CGA CA | Human | 20 |

|  |  |  |  |
| --- | --- | --- | --- |
| **Primer** | **Sequence (5' to 3')** | **Origin** | **Length** |
| *Cd36*-R | TGG AAA TGA GGC TGC ATC TGT | Human | 21 |
| *Cpt1*-F | TGC CCT GAG ACG GGG ATT AT | Human | 20 |
| *Cpt1*-R | GCT TTT CTT TCC AGC CCA GC | Human | 20 |
| *Ppar-α*-F | AGA AGC TGT CAC CAC AGT AGC | Human | 21 |
| *Ppar-α*-R | GAC CAG ATG GTG CTG GTT GT | Human | 20 |
| *Atgl*-F | GTT CCA AAC ACC TCG GTC CT | Human | 20 |
| *Atgl*-R | TCC ACC CAG AGA CAT CTT CCT | Human | 21 |
| *Fasn*-F | GCA AGC TGA AGG ACC TGT CT | Human | 20 |
| *Fasn*-R | AAT CTG GGT TGA TGC CTC CG | Human | 20 |
| *SREBF1*-F | CTG ACC GAC ATC GAA GGT GA | Human | 20 |
| *SREBF1*-R | AAG TGC AAT CCA TGG CTC CG | Human | 20 |
| *ACC1*-F | ATC TTG AGG GCT AGG TCT TTT T | Human | 22 |
| *ACC1*-R | TTC AGC TCC AGA GGT TGG G | Human | 19 |
| *a-SMA*-F | CCG GGA CTA AGA CGG GAA TC | Human | 20 |
| *a-SMA*-R | TTG TCA CAC ACC AAG GCA GT | Human | 20 |
| *Timp1*-F | TGT TGT TGC TGT GGC TGA TAG C | Human | 22 |
| *Timp1*-R | TCT GGT GTC CCC ACG AAC TT | Human | 20 |
| *Pdgfrb* -F | GCC CTT ATG TCG GAG CTG AAG A | Human | 22 |
| *Pdgfrb* -R | GTT GCG GTG CAG GTA GTC CA | Human | 20 |
| *ColⅠα1*-F | GGT GTT CCT GGA GAC CTT GG | Human | 20 |
| *ColⅠα1*-R | AAA CCT CTC TCG CCT CTT GC | Human | 20 |
| *Tgfb1*-F | GGC CAG ATC CTG TCC AAG C | Human | 19 |
| *Tgfb1*-R | GTG GGT TTC CAC CAT TAG CAC | Human | 21 |
| *PAI-1*-F | ACC TCT GAG AAC TTC AGG ATG C | Human | 22 |
| *PAI-1*-R | TCA CCA AAG ACA AGG GCC AG | Human | 20 |
| *FN-1*-F | CCG CCG AAT GTA GGA CAA GA | Human | 20 |
| *FN-1*-R | GGC CCA TGA GAT GGT TGT CT | Human | 20 |
| *GAPDH*-F | AGG TCG GTG TGA ACG GAT TTG | Human | 21 |
| *GAPDH*-R | TGT AGA CCA TGT AGT TGA GGT CA | Human | 23 |
