# Supplementary figures and images for: Paeoniflorin Ameliorates Metabolic Dysfunction-Associated Steatotic Liver Disease by SYK/SH3BP2 Signaling Pathway
Source: Research (Wash D C). 2026 Feb 2;9:1100. doi: 10.34133/research.1100 (PMC12862135; doi:10.34133/research.1100)

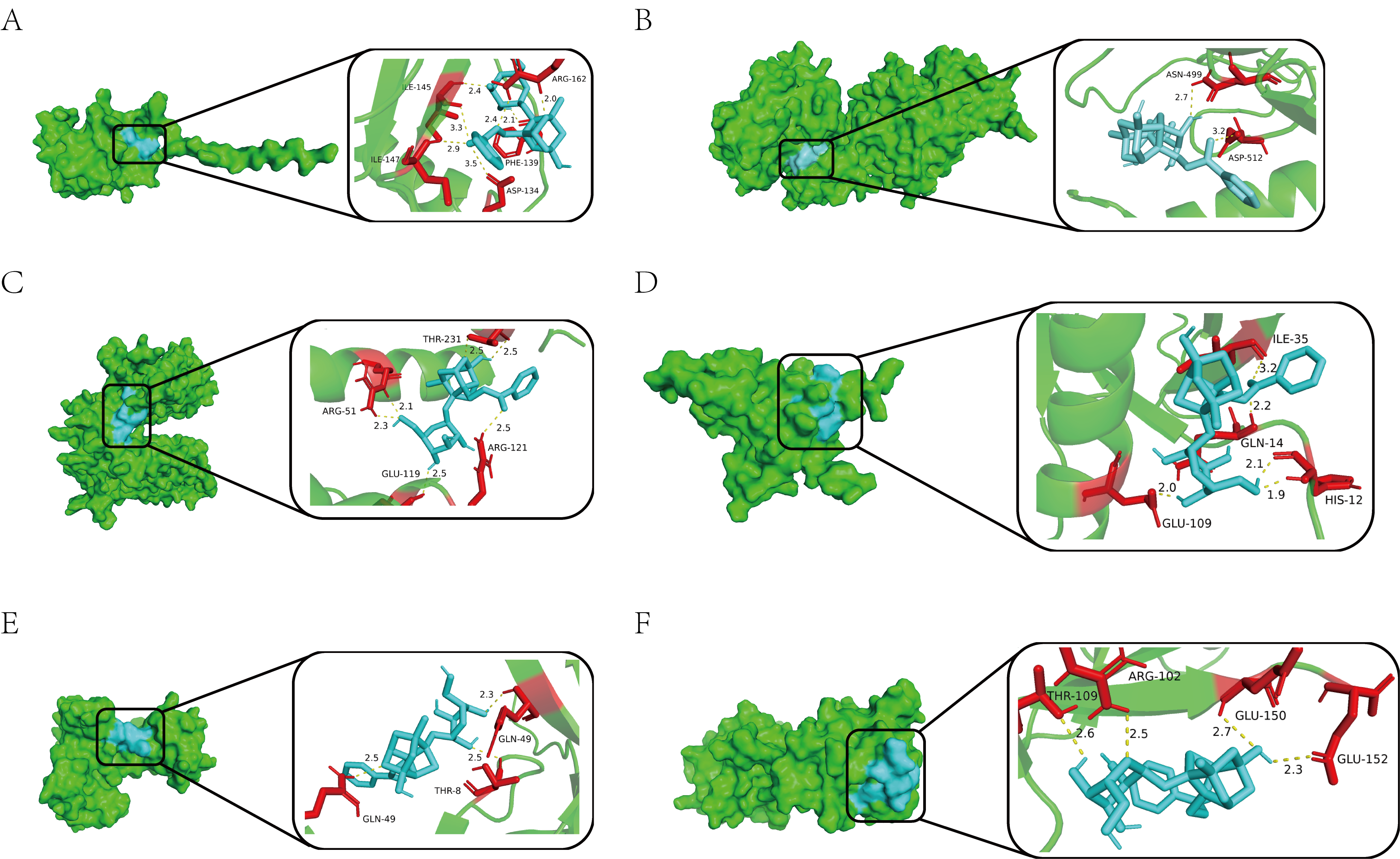

Supplement: Supplementary 1 — Fig. S1 Tables S1 to S4 [file research.1100.f1.zip › Fig. S1/Fig.S1.tif]
